# The Quantification of Vitamin D in Humans: A Promising, Non-Invasive and Cost-Effective Method to Measure 25-Hydroxyvitamin D

**DOI:** 10.3390/biom15040560

**Published:** 2025-04-10

**Authors:** Giulia Squillacioti, Samar El Sherbiny, Veronica Lettico, Federica Ghelli, Marco Panizzolo, Giacomo Scaioli, Manuela Martella, Selene Limoncelli, Giulio Mengozzi, Roberto Bono

**Affiliations:** 1 Department of Public Health and Pediatrics, University of Turin, Via Santena 5 bis, 10126 Turin, Italy; giulia.squillacioti@unito.it (G.S.); samar.elsherbiny@unito.it (S.E.S.); veronica.lettico@edu.unito.it (V.L.); marco.panizzolo@unito.it (M.P.); giacomo.scaioli@unito.it (G.S.); manuela.martella@unito.it (M.M.); roberto.bono@unito.it (R.B.); 2 Clinical Biochemistry Laboratory, Città della Salute e della Scienza di Torino, Molinette Hospital, Corso Bramante 88/90, 10126 Turin, Italy; slimoncelli@cittadellasalute.to.it (S.L.); giulio.mengozzi@unito.it (G.M.); 3 Department of Medical Sciences, University of Turin, Corso Dogliotti 14, 10126 Turin, Italy

**Keywords:** 25(OH)D, vitamin D, serum, urine, saliva

## Abstract

Background: Vitamin D intake and synthesis are essential. Vitamin D deficiency is increasing across all age groups, raising concerns regarding public health. Serum 25(OH)D is measured to define vitamin D deficiency. However, its quantification in non-invasively collected biological matrices is still poorly studied. This study aimed to assess 25(OH)D levels in unconventional matrices using cost-effective analytical methods. Methods: Serum, urine, and saliva were collected from 62 healthy, non-smoking volunteers, 25–44 years of age. Biological samples were analysed using the Enzyme-Linked Immunosorbent Assay (ELISA). The serum was additionally analysed via the chemiluminescent microparticle immunoassay (CMIA), which was used as a benchmark. Results: We observed a linear correlation (Pearson r = 0.44; *p* = 0.05) between the benchmark and ELISA-measured 25(OH)D urinary levels. After stratification by sex, the correlation was stronger and significant only in females (Pearson r = 0.62; *p* = 0.04). Salivary 25(OH)D levels did not correlate with serum levels for both ELISA and CMIA measures. Subjects with a CMIA serum-based deficiency showed lower urinary 25(OH)D levels (*p* = 0.04). Conclusion: Our study opens up the possibility of using urinary 25(OH)D levels as a proxy measurement of vitamin D. Such an approach may allow future investigations on the association between environmental factors and vitamin D assessed in non-invasively collected biological matrices via cost-effective analytical methods.

## 1. Introduction

Vitamin D, or calciferol, is a fat-soluble compound synthesised endogenously by human skin under sunlight irradiation (UVB rays) or obtained exogenously through the ingestion of a limited number of foods, predominantly oil-rich fish, yolks, and fortified products [1]. Vitamin D2, also called ergocalciferol, is derived from diet, while vitamin D3, or cholecalciferol, is produced through dermal synthesis when endogenous 7-dehydrocholesterol (i.e., provitamin D) is exposed to UVB rays [2]. The dermal synthesis of cholecalciferol produces approximately 80% of the total vitamin D circulating in the body. Its synthesis directly depends on the intensity of ultraviolet irradiation and indirectly on outdoor activities and environmental factors that may influence exposure to or the intensity of sunlight. Regardless of the source and form, once inside the body, vitamin D cannot exert its biological effect until it undergoes hydrolysis processes mediated by cytochrome P-450 enzymes (CYPs), including CYP2R1, CYP27A1, and CYP2D25 [3]. The first enzymatic conversion (phase I metabolism) occurs in the liver, where vitamin D is enzymatically converted into 25-hydroxyvitamin D [25(OH)D], which is the major circulating metabolite of vitamin D. Phase II metabolism involves hepatic conjugation reactions involving sulphate ester or a glucuronide moiety, which facilitate renal excretion [4,5]. Once 25(OH)D reaches the kidneys or other organs (e.g., the breasts, colon, brain, etc.), it is further hydroxylated into 1,25-dihydroxyvitamin D, the bioactive form of vitamin D [4], which circulates at much lower concentrations compared to 25(OH)D. Although the biological activity of 24,25-dihydroxyvitamin D (24,25-(OH)2D) is lower than that of 1,25-dihydroxy, it is still a bioactive form [6]. Once converted, 1,25dihydroxyvitamin D exerts its biological effects by binding to the vitamin D receptors (VDRs) expressed in various cell types, including monocytes, macrophages, dendritic cells, etc. [7]. Moreover, VDRs are highly expressed in the small intestine, where vitamin D promotes the absorption of calcium and phosphorus; in bone tissue, where vitamin D promotes osteoblast maturation to osteoclasts; and in the parathyroid glands, which release parathyroid hormone (PTH) to stimulate the renal synthesis of vitamin D in cases of low blood levels. Given its involvement in numerous biological functions, vitamin D deficiency is increasingly recognised as a widespread public health issue affecting all age groups, particularly women and the elderly [8]. Vitamin D deficiency is a major contributor to the development of metabolic bone disorders, such as hypovitaminosis predisposing to osteomalacia (rickets in children) and osteoporosis, which increase the risk of fractures. Additionally, observational epidemiological studies suggest that low vitamin D levels may be associated with the development of acute and chronic diseases, including autoimmune diseases, cardiovascular diseases, diabetes, and certain cancers [9]. Beyond non-communicable diseases, many studies have suggested that vitamin D may reduce the risk of infections, such as influenza and severe acute respiratory syndrome COVID-19 [10]. In 2020, D’Avolio and colleagues observed significantly lower levels of 25(OH)D in PCR-positive patients with coronavirus 2 (*SARS-CoV-2*) compared to negative patients from Switzerland, suggesting that vitamin D supplementation could play a role in preventing the risk of infection [10]. Vitamin D status is typically assessed by measuring serum- or plasma-circulating levels of the total (i.e., bound and unbound) 25(OH)D, which includes both 25(OH)D2 and 25(OH)D3 forms, with the concentration remaining stable for up to three weeks [10]. In the bloodstream, less than 1% of 25(OH)D circulates as a “free” steroid (i.e., unbounded to any proteins) [11]. Most circulating 25(OH)D is bound to a specific vitamin D binding protein (VDBP) and, to a lower extent, to albumin, both of which help regulate the total- and free-circulating levels of vitamin D metabolites [12]. Although there is ongoing debate about the optimal vitamin D level, 25(OH)D levels ranging from 50 to 125 nmol/L (20–50 ng/mL) are generally recommended. The disagreement between the Institute of Medicine (IOM), now called the National Academy of Medicine, and the Endocrine Society (ES) on optimal vitamin D levels stems from differing interpretations of evidence regarding vitamin D’s role in health. According to IOM recommendations, serum 25(OH)D levels below 20 ng/mL (50 nmol/L) are considered inadequate, while the ES considers levels below 30 ng/mL (75 nmol/L) inadequate [13,14]. In addition to the ongoing debate on optimal vitamin D levels, the lack of harmonisation in 25(OH)D quantification methods highlights the need for standardisation in this field to improve the consistency of test results, resolve discrepancies, and enhance the clinical relevance of vitamin D testing in clinical diagnosis, epidemiological studies, and research. Several analytical methods and biological matrices can be used to quantify vitamin D. Liquid chromatography–tandem mass spectrometry (LC-MS/MS) is the gold standard for measuring vitamin D metabolites, offering high sensitivity and accuracy to simultaneously measure vitamin D metabolites such as 25(OH)D2, 25(OH)D3, and 24,25(OH)2D. However, it requires expensive equipment, long analytical times, and specialised personnel, making it less suitable for clinical laboratories. For this reason, immunological assays may be a valid alternative. Many clinical laboratories use automated immunoassays (e.g., Architect, Alinity i, Centaur, Elecsys/Modular/Cobas, Liaison, etc.) [15] and, in recent years, the ELISA (Enzyme-Linked Immunosorbent Assay) technique has gained popularity, especially in research settings [16]. Although competition between the 25(OH)D-specific antibody and VDBP may lead to poor concordance with LC-MS/MS assays [17], their relative ease of use, low cost and small sample volumes make the ELISA a promising method, particularly in research contexts. Measuring vitamin D levels through cost-effective methods in non-invasive biological matrices has become a growing area of interest for researchers and clinicians seeking alternatives to traditional blood sampling. Blood sampling for 25(OH)D concentrations is considered invasive and may cause discomfort, pain, or stress, especially in healthy individuals. Therefore, estimating 25(OH)D concentrations in non-invasively collected biological matrices, such as urine or saliva, would be highly beneficial. Although measuring 25(OH)D in saliva may not always reflect the actual levels in the body [18,19], recent studies have shown a significant positive correlation between serum and salivary vitamin D levels [20,21,22] or, less frequently, no correlation at all [23]. Moreover, since excess 25(OH)D3 is excreted in the urine following a catabolic reaction, measuring vitamin D3 in urine has also been considered potentially useful for vitamin D status determination in the body [24]. Studies have measured urine 25(OH)D levels in nephropathic patients [25], in individuals at risk of developing urinary tract infections or sepsis [26], in pregnant women [27,28], and in healthy adults [29,30]. Although promising, these approaches still require further research to overcome challenges related to sensitivity, standardisation, and correlation with blood levels. Therefore, the present study aims to investigate the feasibility of quantifying 25(OH)D levels through a cost-effective analytical method (ELISA) in non-invasively collected biological matrices (i.e., saliva and urine). Additionally, we evaluated the correlation between ELISA-based measurements of 25(OH)D in serum, urine, and saliva with a benchmark measurement of 25(OH)D assessed in serum via an automated chemiluminescent immunoassay with microparticle capture (CMIA).

## 2. Materials and Methods

### 2.1. Study Design, Setting, and Population

This study was approved by the University of Turin Bioethics Committee (Protocol No. 0654272, 24 October 2023). In September 2023, all necessary documents for obtaining approval from the Bioethics Committee were prepared. At the same time, the feasibility of the study was assessed by informally contacting potential participants among healthcare professionals in the Department of Public Health and Paediatrics at the University of Turin in Italy to gauge their willingness to participate in the study. Recruitment began on 26 October 2023, following the approval of the Bioethics Committee.

Subjects were considered eligible if they met the following criteria: if they were aged between 25 and 45 years, non-smokers, not pregnant, had taken no vitamin D supplementation within the last month, lacked any history of specific diseases (i.e., nephropathies, hepatopathies, coeliac disease, Crohn’s disease, hyperparathyroidism, psoriasis, and arthritis), and did not take antiepileptics, glucocorticoids, antiretrovirals, antimycotic, cholestyramine, or orlistat. These criteria were intended to increase the homogeneity of the sample population by reducing factors that could influence circulating vitamin D levels.

Eligible volunteers were recruited from 26 October 2023 to 3 November 2023 at the Laboratory of Environmental and Occupational Hygiene of the Department of Public Health and Paediatrics of the University of Turin. After signing the informed consent form, each participant provided fasting biological samples (i.e., blood, saliva, and urine). Procedures were performed in the early morning (7.30–9.30 a.m.). Additionally, participants were asked to complete an online questionnaire designed to gather information on their lifestyle habits and characteristics that might impact vitamin D status.

### 2.2. Biological Sample Collection, Processing, and Storage

Each subject provided a blood sample (8.5 mL Vacutainer^®^ tube with separator gel, BD Franklin Lakes, NJ, USA), a drooled saliva sample (minimum 2 mL, collected in a 50 mL Falcon^®^ Corning, Glendale, AZ, USA), and a spot urine sample (a few millilitres in a sterile 200 mL container with a screw cap). The medical personnel performing the procedures wore protective equipment and followed aseptic techniques. All biological samples were processed as detailed below and then stored at −80 °C until laboratory analysis, which was within 1 month.

To minimise risks and discomfort for participants, blood sample collection was conducted according to updated recommendations [31]. To obtain serum, whole blood was left at room temperature to clot and, within 1 h, was centrifuged at 3000 rpm for 10 min and was then aliquoted and stored. Participants provided a minimum of 2 mL of unstimulated saliva using passive drool, abstaining from tooth brushing, eating, and drinking for 30 min before sample collection. Whole saliva samples were centrifuged at 3000 rpm per 10 min, and the supernatant was aliquoted and stored. After provision, urine samples were centrifuged at 3000 rpm per 10 min, and the supernatant was aliquoted and stored.

### 2.3. 25(OH)D Quantification

25(OH)D levels were quantified in serum samples by an automated CMIA (Alinity i. Abbott Ireland Diagnostics Division Lisnamuck, Longford Co., Longford, Ireland), which is commonly used in the Clinical Biochemistry Laboratory (Città della Salute e della Scienza, Turin, Italy) of the Molinette Hospital to diagnose vitamin D deficiency. This measure was used as a benchmark for comparisons with the following quantifications.

Serum, saliva, and urine 25(OH)D levels were quantified using an ELISA kit and following the manufacturer’s instructions (My BioSource, MBS268910, San Diego, CA, USA). Depending on the biological matrix, specific dilution factors were applied, which were determined based on several preliminary analyses (i.e., spike-and-recovery and linearity-of-dilution experiments ). According to the Consensus Statement on Vitamin D Status Assessment and Supplementation [32], both units of measure (mol/L and ng/mL) were reported, and urine levels were corrected to the excretion rate, thus expressed as the urine creatinine ratio (ng/mg creatinine).

### 2.4. Other Covariates and Potential Confounders

A self-administered online questionnaire was used to collect key information on lifestyle habits and characteristics potentially impacting vitamin D status (i.e., potential confounders). In particular, adherence to the Mediterranean diet (AMD) was evaluated using the Medi-Lite standardised questionnaire, validated in 2017 [33]. The final score, from 0 (low adherence) to 18 (high adherence), was divided into tertiles describing low, medium and high AMD (the 1st, 2nd, and 3rd tertiles, respectively); details on demographics, exposure to passive smoking, anthropometric data (i.e., weight and height) and habits potentially influencing vitamin D levels (e.g., phototype, sun creams use, exposure to the sun in the last weeks, etc.) were collected using additional free questions. Self-reported weight and height were used to calculate Body Mass Index (BMI) according to the following formula: weight (kg)/height^2^ (metres).

### 2.5. Statistical Analysis

Statistical analysis was performed using the Stata (StataCorp. 2024. Stata Statistical Software: Release 18. College Station, TX, USA: StataCorp LLC.), and figures were generated using SPSS (IBM Corp. Released 2023. IBM SPSS Statistics for Windows, Version 29.0.2.0 Armonk, NY, USA: IBM Corp). Quantitative variables were summarised using the mean and standard deviation (SD) or, if skewed, the median and interquartile range (IQR), while qualitative variables were presented using absolute frequency values (number, n) and relative frequencies (%). Between-group differences in 25(OH)D levels were tested using a t-test for normally and homoscedastic distributed variables. Otherwise, we opted for one of the following non-parametric tests. The Mann–Whitney U-test was used to test the difference between the groups defined by dichotomous variables (e.g., males vs. females; exposed to passive smoking vs. not exposed, etc.). The Kruskal–Wallis H-test was used for groups defined by the categorical variable with more than two categories (e.g., low, medium or high AMD, etc.). χ^2^ or Fisher’s exact test was used if the absolute frequencies in each category were statistically different. A correlation analysis (Pearson’s r) was performed to test the linear relationship between 25(OH)D levels in different biological matrices and was quantified using the ELISA or CMIA analytical method.

For sensitivity analyses, we firstly stratified the correlation analysis by sex and secondly compared 25(OH)D medians (Kruskal–Wallis test) across the status of different vitamin D levels (deficiency vs. sufficiency), which are defined according to recommendations provided by the Institute of Medicine [14] and the Endocrine Society [13]. The significance level was set at 5%.

## 3. Results

### 3.1. Sample Population Characteristics

As reported in Table 1, a sample of 62 young people (53.2% females) aged between 25 and 44 years (31.5 ± 5.1 years) was enrolled. Most participants were normal-weight adults (71%), around 21% were overweight, and none were obese. Around 52% of the study sample were workers, and 35.5% were students, but they shared similar working conditions as they had been recruited among health professionals and residents of a university department. In line with the inclusion criteria, none were active smokers, while around 15% of the participants reported exposure to passive smoking. According to the scoring obtained with the Medi-Lite questionnaire, only 22.6% of the enrolled subjects sample reported a high AMD. Concerning sun exposure-related behaviours, 73% of the participants routinely used sun cream, although most of them (54.8%) declared short habitual sun exposure periods lasting for less than 15 min per day. Regarding phototypes, participants with very light skin (phototype I) or light skin and brown hair (phototype II) made up the largest proportion of the sample, accounting for around 36% and 37% of the subjects, respectively.

### 3.2. Serum 25(OH)D Quantification by CMIA (Benchmark Measure)

Serum 25(OH)D levels, quantified by the CMIA, are reported in Table 2. As previously mentioned, this measurement was considered the benchmark in our study because the CMIA method is commonly and officially used to diagnose vitamin D deficiency. Frequencies of deficiency greatly differed according to IOM and ES cut-offs, with the latter classifying around 69% of the subjects as deficient. No differences were detected among CMIA-assessed serum levels of 25(OH)D between males and females (*p* = 0.70) or discrepancies among deficiency frequencies according to IOM or ES (*p* = 0.76 and 0.73, respectively).

### 3.3. Serum, Urine, and Saliva 25(OH)D Quantification by ELISA

Table 3 summarises the 25(OH)D levels assessed in serum, saliva, and urine by ELISA. Median 25(OH)D levels in urine were higher in females compared with those measured in males (*p* = 0.04). Between-group comparisons highlighted lower median 25(OH)D levels in saliva in participants reporting exposure to passive tobacco smoking (13.1 ng/mL) compared to those not passively exposed (19.2 ng/mL) (Mann–Whitney U-test *p*-value = 0.02). No other statistically significant differences were detected in 25(OH)D levels quantified in all biological matrices and both analytical methods.

### 3.4. Correlation Between the Benchmark Measure and 25(OH)D, Measured by ELISA in Serum, Urine, and Saliva

As reported in Table 4, urine 25(OH)D levels quantified by ELISA were significantly and positively correlated with the benchmark measure of vitamin D and serum 25(OH)D quantified by CMIA (Pearson’s r = 0.44, *p* = 0.05). No other correlations were observed among different matrices and analytical methods.

**Figure 1:**
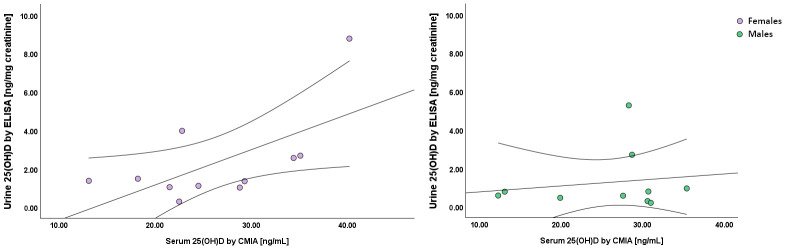
Correlation between urine and serum 25(OH)D (n = 21) measured by Enzyme-Linked Immunosorbent Assay (ELISA) and chemiluminescent immunoassay with microparticle capture (CMIA) methods, stratified by sex.

The same results were observed after sex stratification (Figure 1). However, the statistical significance was only confirmed in women whose urine 25(OH)D levels were more strongly correlated with the benchmark quantification (Pearson’s r = 0.62; *p* = 0.04).

**Figure 2:**
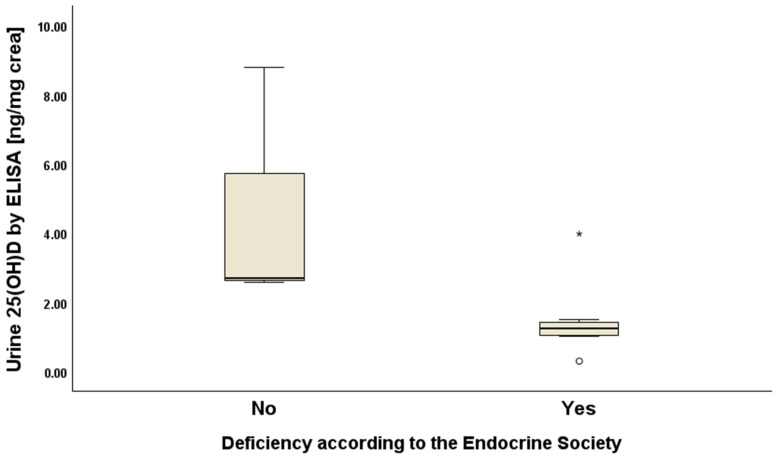
Urine 25(OH)D levels measured by Enzyme-Linked Immunosorbent Assay (ELISA) according to vitamin D deficiency status in females. The asterisk represents values that are >3.0 IQR above the third quartile, while the circle represents values that are <1.5 IQR below the first quartile.

Additionally, we further analysed if urine 25(OH)D levels varied by vitamin D deficiency status and observed that females with a Vitamin D deficiency, according to the ES, had lower median urine 25(OH)D (1.24 ng/mg creatinine) compared to those without deficiency (2.69 ng/mg creatinine) (Mann–Whitney U-test *p*-value = 0.04) (Figure 2). We did not observe the same results for men (*p*-value = 0.39) or in subjects classified according to the IOM cut-offs.

## 4. Discussion

The present study suggests a positive and statistically significant linear correlation between 25(OH)D levels, measured using a cost-effective method (ELISA) in non-invasive samples (urine), and a benchmark measure of vitamin D status (i.e., serum 25(OH)D quantified by CMIA). After stratifying by sex, this relationship was confirmed only in women and not in men. Additionally, we observed that median urine 25(OH)D levels were lower in women classified as vitamin D-deficient based on the ES threshold applied to serum benchmark quantifications. Serum and saliva 25(OH)D levels, quantified by ELISA, were not correlated with the benchmark measure or with each other.

Median serum 25(OH)D levels, quantified by CMIA, were comparable, although slightly higher, compared to those reported by Lahoz and colleagues [34], who measured 25(OH)D using the same analytical method in a group of individuals without vitamin D supplementation. The vast majority of previous studies employed a wide range of analytical techniques to assess serum 25(OH)D levels, mainly including ELISA [20,22,23,27,35,36,37,38,39], electrochemiluminescence enzyme immunoassays [18,39,40,41], chromatographic techniques [29,42,43,44], radioimmunoassay [45], and other techniques [19,30]. In these studies, the reported average/median serum 25(OH)D levels varied widely and were only partly comparable to our results, particularly those obtained from chromatographic or radioimmunoassay techniques, which ranged from 18.7 to 32.8 ng/mL and from 24.7 to 36.4 ng/mL, respectively. In our study, when applying the IOM and ES recommendations to categorise the distribution of serum 25(OH)D levels derived from CMIA measurements, 25.8% and 67.7% of participants were classified as vitamin D-deficient, respectively. The observed prevalence of deficiency was lower than the age-specific (19–45 years) and latitude-specific prevalence reported by Cui and colleagues in their systematic analyses, which included more than 150 studies [46]. Similarly, compared with the deficiency prevalence observed in the Italian adult population (i.e., 33% in the study by Lippi and colleagues [47]; 36% in the study by Lippi and colleagues [48]), we observed a lower deficiency prevalence. Another Italian study on a sample of 18,131 Italians from the general population reported a vitamin D deficiency prevalence of 35.47%, with a significantly higher prevalence in men than in women (40.12% vs. 33.10%) [49]. While the deficiency prevalence reported by Bani and colleagues was higher than the one observed in our study, their finding of a higher prevalence in men than in women is consistent with our results.

To date, the quantification of 25(OH)D in urine has been scarcely studied. In general, urinary vitamin D levels are quantified by chromatographic techniques [24,28,29], while some authors have reported measurements via immunoassays [24,28,29], and some authors have reported measurements using immunoassays [25,26] and the NLucVDR assay system [29,30]. Salivary 25(OH)D quantifications are more frequent than urinary quantifications and are typically performed using ELISA techniques, with reported results ranging widely from 36 pg/mL to 85.7 ng/mL. [21,23]. The mean salivary values observed in our study (i.e., 18.8 ng/mL) closely align with those reported by Sari et al. (i.e., 16.54 ng/mL) [22], obtained from a sample of 56 healthy Indonesian adults aged 18 to 60 years.

Our main finding was a statistically significant and positive correlation between serum benchmark 25(OH)D levels and urinary ones. This result is inconsistent with the findings from Kushioka and colleagues [29,30], who suggested that urine analysis may be useful for predicting serum 25(OH)D_3_ levels and developed a specific analytical method, the NLucVDR assay. Similarly, a recent research letter highlighted that urine analysis could also serve as a diagnostic tool for vitamin D detection, particularly in aiding clinicians in managing urinary tract infections [26]. In contrast, we did not observe correlations between vitamin D levels in serum and saliva. This finding aligns with some previous studies [19,23], but it is in contrast with others [18,20,21,38]. Notably, the detected correlation was based on a limited number of observations, as only around 34% of the urine samples (n = 21) had 25(OH)D levels above the limit of detection (LOD).

Regardless of the biological matrix, women, on average, exhibited higher 25(OH)D levels than men. This finding is consistent with previous research on sex-specific vitamin D levels involving adults from Saudi Arabia [50], Chile [51], and India [52] but contrasts with studies reporting a greater vulnerability to vitamin D deficiency in women [46,53,54,55]. Overall, sex appears to play a crucial role in the differences in circulating 25(OH)D levels between men and women, as well as in the degree of correlation between serum and urine 25(OH)D measurements. Reproductive hormones may affect the expression of genes related to vitamin D metabolisms [56] due to estrogenic therapies [57]. Previous studies have reported higher total 25OHD concentrations in women who take oral contraceptives containing oestrogen, possibly because of an oestrogen-related increased hepatic hydroxylation of vitamin D [58], although this phenomenon was not confirmed in girls not taking hormonal contraceptives [59]. In addition, VDBP concentration is generally higher in women than in men [60], and, together with the hormone asset, this element may partly explain the higher vitamin D levels observed in young women. Furthermore, evidence from in vivo studies suggests that when compared to male mice, female mice can synthesise vitamin D in their skin more efficiently [61]. On the whole, sex-dependent influences on vitamin D levels and interplay with other molecules and/or therapies seem to be critical [62] and should always be taken into account.

In this study, we did not observe any correlation between serum 25(OH)D concentration measured by ELISA and serum CMIA. Although seemingly surprising, this result suggests that a matrix effect could have strongly influenced the ability of the binding agent to efficiently associate with 25(OH)D [63], endorsing the need for the improvement of the analytical method by adding purification or precipitation steps. As one of the main limitations, we did not apply any pre-treatment step. This should be intended as an important step for future research on this topic, particularly regarding urine samples with 25(OH)D concentrations that might be very low, thus requiring additional method optimisation, both in terms of deconjugation and the breakdown of the carrier proteins’ bond. Given the explorative nature of the present study, we opted for a simpler first approach that, given the promising results, deserves future in-depth implementations. Another potential limitation is the sample size, which, however, was extremely homogeneous in terms of several potential confounders (e.g., age, sex, education level, ponderal status, etc.). Moving on to the strengths, our study is one of the few existing works on vitamin D quantification in emerging biological matrices such as urine and saliva. Two analytical methods (ELISA and CMIA) were used, and the correlation among their results was investigated in a sample of young adults.

## 5. Conclusions

Although far from definitive, our findings suggest the potential usefulness of non-invasively collected biological matrices, particularly urine, as potential novel media for quantifying 25(OH)D in young adults. The moderate correlation observed between 25(OH)D measured in urine (via ELISA) and serum (via CMIA) supports the feasibility of using non-invasive, cost-effective, and time-efficient analyses to advance vitamin D research. This approach is especially relevant for environmental epidemiology studies, which require higher subject compliance rates. Upon further validation, the quantification of 25(OH)D levels in urine could serve as a useful proxy for vitamin D status, particularly in non-diagnostic settings. It could be employed to explore the interaction between environmental and behavioural factors, such as air pollution and lifestyle habits, including smoking exposure and diet. Given that vitamin D deficiency is considered a contributing factor to various human diseases, supporting future epidemiological investigations into environmental and behavioural factors is essential. This would indirectly enhance our understanding of environmental exposures and chronic diseases. While this study provides preliminary insights, several aspects remain unexplored. Future research should focus on further improving the ELISA analytical methods, particularly through the evaluation of (1) conjugated and unconjugated 25(OH)D in urine; (2) free and total 25(OH)D levels in all biological matrices; and (3) potential cross-reactivity with other 25(OH)D metabolites.

## Figures and Tables

**Table 1:** Sample population characteristics.

Characteristics	Overall 62 (100%)	Females 33 (53.2%)	Males 29 (46.8%)
Age, mean (SD) [years]	31.5 (5.1)	30.4 (4.1)	32.8 (5.9)
BMI categories, n%			
Normal weight	44 (71.0)	30 (48.4)	14 (22.6)
Overweight	13 (20.9)	2 (3.2)	11 (17.7)
Missing data	5 (8.1)	1 (1.6)	4 (6.5)
Occupation, n (%)			
Students	22 (35.5)	12 (19.4)	10 (16.1)
Workers	32 (51.6)	18 (29.0)	14 (22.6)
Missing data	8 (12.9)	3 (4.8)	5 (8.1)
AMD, n (%)			
Low (1st tertile)	34 (54.8)	20 (32.3)	14 (22.6)
Medium (2nd tertile)	9 (14.5)	6 (9.7)	3 (4.8)
High (3rd tertile)	14 (22.6)	6 (9.7)	8 (12.9)
Missing data	5 (8.1)	1 (1.6)	4 (6.5)
Tobacco smoking exposure, n (%)			
Passive smokers	9 (14.5)	4 (6.5)	5 (8.1)
Missing data	5 (8.1)	1 (1.6)	4 (6.5)
Sun exposure behaviour, n (%)			
Sun cream users	45 (72.6)	26 (41.9)	19 (30.7)
Missing data	5 (8.1)	6 (9.7)	4 (6.5)
Sun exposure < 15 min/day	34 (54.8)	22 (35.5)	12 (19.4)
Missing data	8 (12.9)	2 (3.2)	6 (9.7)
Phototype, n (%)			
I. very light skin	22 (35.5)	15 (24.2)	7 (11.3)
II. light eyes and skin	23 (37.1)	11 (17.7)	12 (19.4)
III. light skin and brown hair	5 (8.1)	2 (3.2)	3 (4.8)
IV. olive skin	7 (11.3)	4 (6.5)	3 (4.8)
Missing data	5 (8.1)	1 (1.6)	4 (6.5)

SD = standard deviation; BMI = Body Mass Index; AMD = adherence to the Mediterranean Diet.

**Table 2:** Serum 25(OH)D quantification by CMIA (benchmark measure).

Vitamin D Benchmark Levels (CMIA)	Overall 62 (100%)	Females 33 (53.2%)	Males 29 (46.8%)	*p*-Value
Serum 25(OH)D, mean (SD)				
ng/mL	26.1 (9.1)	26.6 (9.6)	25.6 (8.7)	0.70 ^a^
nmoL/L	65.3 (22.7)	66.4 (23.9)	64.1 (21.7)	0.70 ^a^
Serum 25(OH)D, median (IQR)				
ng/mL	25.2 (19.9–31.2)	24.5 (20.8–32)	25.2 (19.9–35.7)	0.84 ^b^
nmoL/L	62.9 (49.8–78.0)	61.3 (52–80)	63 (49.8–77.5)	0.84 ^b^
Vitamin D deficiency, n (%)				
IOM	16 (25.8)	8 (12.9)	8 (12.9)	0.76 ^c^
ES	42 (67.7)	23 (37.1)	19 (30.6)	0.73 ^c^

CMIA = chemiluminescent immunoassay with microparticle capture; SD = standard deviation; IOM = Institute of Medicine recommendations; ES = Endocrine Society recommendations. ^a^ *t*-test or ^b^ Mann–Whitney and ^c^ χ^2^ were used to test differences between females and males.

**Table 3:** ELISA quantifications of 25(OH)D in serum, saliva, and urine.

Vitamin D ELISA Levels	Overall 62 (100%)	Females 33 (53.2%)	Males 29 (46.8%)	*p*-Value
Serum 25(OH)D, mean (SD)				
ng/mL	6.54 (1.59)	6.89 (3.31)	6.13 (1.80)	0.06
nmoL/L	16.34 (3.99)	17.24 (3.31)	15.32 (4.50)	0.06
Saliva 25(OH)D, mean (SD)				
ng/mL	18.79 (6.21)	18.89 (6.44)	18.68 (6.10)	0.89
nmoL/L	46.98 (15.53)	47.23 (16.10)	46.69 (15.14)	0.89
Urine 25(OH)D, median (IQR)				
ng/mg creatinine	1.05 (2.57–0.59)	1.38 (2.69–1.05)	0.69 (0.96–0.47)	0.04
nmoL/mmoL creatinine	0.28 (0.68–0.18)	0.44 (0.78–0.28)	0.20 (0.26–0.14)	0.04

ELISA = Enzyme-Linked Immunosorbent Assay; SD = standard deviation; IQR = interquartile range. *t*-test and Mann–Whitney were used to test differences between females and males.

**Table 4:** Correlation between benchmark 25(OH)D levels and those measured by ELISA in serum, saliva, and urine.

25(OH)D	Serum Benchmark (CMIA) [ng/mL]	Serum ELISA [ng/mL]	Urine ELISA [ng/mg Creatine]
Serum ELISA [ng/mL]	r = −0.01		
	*p* = 0.94		
	n = 62		
Urine ELISA [ng/mg crea]	r = 0.44	r = 0.11	
	*p* = 0.05	*p* = 0.63	
	n = 21	n = 21	
Saliva ELISA [ng/mL]	r = 0.10	r = −0.12	r = 0.05
	*p* = 0.43	*p* = 0.36	*p* = 0.83
	n = 62	n =62	n = 21

ELISA = Enzyme-Linked Immunosorbent Assay; CMIA = chemiluminescent immunoassay with microparticle capture. All *p*-values are derived from Pearson’s correlation analysis.

## Data Availability

The datasets presented in this article are not readily available because some restrictions to the dataset access have been declared in the documents approved by the University of Turin’s Bioethical Committee. Requests to access the datasets should be directed to giulia.squillacioti@unito.it.
